# DEcompressive Surgery for the Treatment of malignant INfarction of the middle cerebral arterY - Registry (DESTINY-R): design and protocols

**DOI:** 10.1186/1471-2377-12-115

**Published:** 2012-10-02

**Authors:** Hermann Neugebauer, Peter U Heuschmann, Eric Jüttler

**Affiliations:** 1 Center for Stroke Research Berlin (CSB), Charité - Universitätsmedizin Berlin, Augustenburger Platz 1, 13353, Berlin, Germany; 2 Institute for Clinical Epidemiology and Biometry, Center for Clinical Studies, and Comprehensive Heart Failure Center, University of Würzburg, Petrinistr. 33a, 97080, Würzburg, Germany

**Keywords:** Decompressive surgery, Hemicraniectomy, Ischaemic stroke, Malignant MCA infarct, Registry

## Abstract

**Background:**

Randomized controlled trials (RCT) on the treatment of severe space-occupying infarction of the middle cerebral artery (malignant MCA infarction) showed that early decompressive hemicraniectomy (DHC) is life saving and improves outcome without promoting most severe disablity in patients aged 18–60 years. It is, however, unknown whether the results obtained in the randomized trials are reproducible in a broader population in and apart from an academical setting and whether hemicraniectomy has been implemented in clinical practice as recommended by national and international guidelines. In addition, they were not powered to answer further relevant questions, e.g. concerning the selection of patients eligible for and the timing of hemicraniectomy. Other important issues such as the acceptance of disability following hemicraniectomy, the existence of specific prognostic factors, the value of conservative therapeutic measures, and the overall complication rate related to hemicraniectomy have not been sufficiently studied yet.

**Methods/Design:**

DESTINY-R is a prospective, multicenter, open, controlled registry including a 12 months follow-up. The only inclusion criteria is unilateral ischemic MCA stroke affecting more than 50% of the MCA-territory. The primary study hypothesis is to confirm the results of the RCT (76% mRS ≤ 4 after 12 months) in the subgroup of patients additionally fulfilling the inclusion cirteria of the RCT in daily routine. Assuming a calculated proportion of 0.76 for successes and a sample size of 300 for this subgroup, the width of the 95% CI, calculated using Wilson's method, will be 0.096 with the lower bound 0.709 and the upper bound 0.805.

**Discussion:**

The results of this study will provide information about the effectiveness of DHC in malignant MCA infarction in a broad population and a real-life situation in addition to and beyond RCT. Further prospectively obtained data will give crucial information on open questions and will be helpful in the plannig of upcomming treatment studies.

**Trial registration:**

(ICTRP and DRKS): DRKS00000624

## Background

Severe space-occupying middle cerebral artery infarction (malignant MCA infarction) is a worst case scenario in stroke medicine. Comparable to other devastating subtypes of stroke such as severe intracranial hemorrhage and basilar artery thrombosis therapeutic options are limited and questionable with respect to improved outcome. Even under maximum intensive care treatment most patients die after only few days due to relentless edema formation, brain tissue shift and transtentorial herniation [1-3]. In contrast, decompressive hemicraniectomy (DHC) has been proven a life saving surgical intervention. Its efficacy in malignant MCA infarction has been demonstrated in three randomized controlled trials (RCT), their a pooled-analysis, and a recent meta-analysis: Early DHC within 48 hours significantly reduces mortality and improves the chance of surviving with mild to moderate disability without increasing the risk of most severe disability [4-7].

Although the RCT and the two analyses gave answer to the most pertinent question, they were not powered for other important issues: First, due to the strict inclusion criteria of the RCT, only healthy and independent young patients (18–60 years) were treated. In addition, a positive treatment effect was only found within 48 hours after stroke onset [4-7]. Therefore, it is unclear if the results can be extrapolated to real world situations, when co-morbidities and dependency are common and the decision to proceed with decompressive surgery is made later on in the course of the disease. Second, since the RCT were not powered for adequate subgroup analysis - only 109 patients were included in the latest meta-analysis, 58 underwent DHC and 51 were treated conservatively [4]- it remains unclear which individual patients benefit from surgical decompression, e.g. with respect to age, presence of aphasia, and timing of surgical intervention. Moreover, it remains unclear which clinical or radiolgical factors sufficiently predict edema formation and thus allow selection of patients for DHC in advance of clinical deterioration [8-12]. Third, with respect to the extent of the infarction and the unrealistic expectation of complete recovery after severe stroke, “favorable” outcome was defined as a score of ≤ 4 on the modified Rankin scale (mRS) in the pooled-analysis of the RCT [7]. It is a matter of ongoing debate amongst experts if such a condition may be classified as “favorable” [13]. Indeed, some authors and the latest meta-analysis defined favorable as a mRS of ≤ 3 [4]. Other authors think that the question of favorable (or in such severe diseases rather “acceptable”) outcome should be answered by the patients themselves. Unfortunately, the contradictory data on quality of life and retrospective consent to hemicraniectomy after malignant MCA infarction that are currently found in the literature are of little help in preoperative decision making [5,9,14-18]. Fourth, the actual effectiveness of conservative treatment has never been addressed in detail, e.g. the RCT showed different outcomes in the conservative treatment groups: patients who were treated on an intensive care unit [5] had a higher rate of survival (47% vs. 22%) and more often better functional outcome (mRS 0–4: 33% vs. 0%) than patients treated on a stroke unit or a general ward [6]. Fifth, despite the life-saving character of DHC, the overall case fatality rate in patients who underwent surgery in the RCT was still 29% [7]. This means that nearly every third patient dies for reasons not well understood. Similarly, no valid data are available on the actual rate of complications especially concerning re-implantation of the skull.

Most of these issues are a commonplace in the scientific community and have been addressed in a number of case series and non-randomized case control studies [19-21]. However, most of these studies are retrospective in nature, lack appropriate control groups, and show heterogeneous or inconclusive results. Therefore, proper investigation in a large-size, prospective study is urgently needed. Besides, the implementation of hemicraniectomy in clinical practice has not been reviewed since the publication of the RCT.

## Methods

### Study design

DESTINY-R is a prospective, multicenter, open, and controlled registry. There will be no active allocation of the patients either to hemicraniectomy or any conservative treatment option. At the time of publication 45 neurologic and neurosurgical departments were registered as actively recruiting study centers. All participating centers have adequate experience with the management of acute ischemic stroke, intensive care treatment of patients with increased intracranial pressure (ICP) and access to neurosurgical facilities on a 24-hours/day basis.

### Ethics

The study protocol and all subsequent amendments are approved by the leading ethic committee of the Charité- Universitätsmedizin Berlin (Ethic Committee at Campus Benjamin Franklin, Reference number EA4/108/109, date of approval 12/08/09) and the local ethics committees of the participating centers. The study is performed in accordance with the Declaration of Helsinki and its subsequent amendments, as well as the guidelines of Good Clinical Practice. The study is registered at the German Clinical Trial Register (DRKS) and the International Clinical Trials Registry Platform (ICTRP). The registration number is DRKS00000624. Written informed consent is obtained from subjects who meet the study inclusion criteria or their legal representatives, respectively.

### Study subject recruitment

At each study site patients older than 18 years of age are screened and asked for participation if they meet the following inclusion criteria: (a) clinical signs of unilateral MCA infarction, (b) ischemic infarction affecting at least 50% of the MCA territory confirmed by computed tomography (CT) or magnetic resonance imaging (MRI). Additional involvement of the anterior and/or posterior cerebral artery territories may be present, (c) written informed consent of the patient or their legal representative. Because treatment is independent from participation in DESTINY-R there is no time interval for inclusion. The only exclusion criterion is a simultaneous or contemporary acute brain injury, e.g. traumatic brain injury or concomitant infarction contralateral or infratentorial in addition to the index- infarction.

### Study protocol

After written informed consent is obtained patients are enrolled in the study and baseline data are documented (visit 1). Subsequently, patient’s medical treatment data are recorded throughout the regular treatment. No additional examinations or interventions are carried out by reasons of the study. At discharge medical treatment data are documented in the case-report-form (visit 2). One year after symptom onset patients or their legal representatives are contacted by phone or letter (visit 3). Follow-up data are also documented in the CRF. After completion a copy of the CRF and a compact disc containing the patients’ pseudonymized neuroimaging data is sent to the coordinating center (Center for Stroke Research Berlin (CSB)), which is responsible for data management and analysis.

### Data collection

Baseline documentation of clinical data includes demographic factors, past medical history, history of present illness, and the previous functional status measured on the mRS and NIHSS. Imaging data includes infarct localization, presence of space-occupying edema, and hemorrhagic transformation. Considering conservative treatment measures, the use of ICP-lowering drugs, and data on sedation, muscle relaxation and mechanical ventilation will be recorded. With respect to surgical procedures, the time-to-surgery, the diameter of craniotomy, and the type of duraplasty will be recorded. Complications during the initial hospital stay will also be recorded. At follow-up, data acquisition is done using a structured interview including the modified Rankin Scale, the Barthel-Index, the EuroQoL-5D, the SF-36, and the Hamilton Depression Rating Scale (HDRS). Furthermore, complications during the follow-up period and, if applicable, the retrospective consent to treatment will be recorded.

### Outcome measures

The primary outcome measure is functional outcome as determined by the mRS score, dichotomized between 0–4 and 5 or 6 at 1 year ± 14 days after symptom onset in the subgroup of patients additionally fulfilling the inclusion criteria of the RCT. Secondary outcome measures are: (a) mortality, (b) median time of survival assessed by the Kaplan-Meier estimator, (c) functional outcome as determined by the mRS Score dichotomized between 0–3 and 4–6 at 1 year ± 14 days after symptom onset, (d) treatment modes (i.e. osmotherapy, hyperventilation, sedation, etc.), assessed at discharge if applicable, time to decompressive surgery, diameter of bone flap/craniectomy measured in situ and by neuroimaging, type of duraplasty, if applicable, as well as complications (i.e. hygroma, impaired wound healing, epidural and subdural hematoma, parenchymatous hematoma, wound and bone flap infections) and (f) time to cranioplasty assessed 1 year ± 14 days after symptom onset if applicable, other complications (i.e. pneumonia, and any other infection, thrombosis, seizures), (g) quality of life determinded by the SF-36 and EuroQoL-5D questionnaires and depression assessed by the HDRS, and (h) rate of retrospective consent to treatment assessed 1 year ± 14 days after symptom onset.

### Sample size calculation

Assuming a calculated proportion of 0.76 for successes in the subgroup of patients additionally fulfilling the inclusion cirteria of the RCT, and a sample size of 300 for this subgroup, the width of the 95% CI, calculated using Wilson's method, will be 0.096 with the lower bound 0.709 and the upper bound 0.805 [22]. Given an estimated enrolement of 4 patients per center/year at 45 study centers plus one year of follow-up, data acquisition would be concluded in 3 years. Thus the end of the study is planned for 2014.

### Data analysis

All outcome measures will undergo descriptive data analysis, including mean, standard deviation, median, range, as well as absolute and relative frequencies depending on the scales of the variables. Additionally, descriptive *p*-values for group comparison and the corresponding 95% confidence interval will be indicated. Two-sample *t* testing, the Mann–Whitney *U*-test, or *χ*2-testing will be used to analyze group differences as applicable. Kaplan-Meier estimates and log-rank statistics will be used for assessing cumulative risk of death at 12 months. The comparability of different treatment groups will be depicted by comparing demographic and baseline data. Statistical analysis will be performed using the latest SAS software version (SAS Institute Inc., Cary, NC).

### Monitoring

Internal and external audits will be held in order to assure quality standards according to the Declaration of Helsinki, ICH-GCP guidelines, and governmental standards.

### Publication of the trial results

The trial results will be published independently of the results by the members of the Steering Committee and the participating centers (DESTINY-R study group). Authorship as well as publication of partial results will be regulated by the Steering Committee.

## Discussion

We advocate the use of a prospective register to further characterize patients with malignant MCA infarction and evaluate implementation, effectiveness and adverse events of current treatment concepts, including DHC. Although first trends in prevalence and outcome of DHC are promising and seem comparable to the results of the RCT [23,24], the use of a large database allows the representation of a much broader population of patients treated for malignant MCA infarction. The anticipated sample size should allow confirming the results of the RCT and raising new hypotheses even if the population turns out to be more heterogeneous than expected. The planned inclusion of at least 300 patients in a reasonable time frame can only be achieved in a multicenter approach. The open design allows continuing initiation of cooperative study centers. The very conservative estimate of 4 patients/year per center would probably allow extending patient recruitment to 500 or even 1000 if certain questions would need a larger sample size to be investigated. However, due to the nature of a prospective registry, only hypotheses may be created, which then need further investigation in RCT.

To avoid the investigation of a very restricted group of patients, the inclusion criterion of infarct size was intentionally set comparatively wide. Thus any patient with a MCA infarction involving more than 50% of the territory can be included irrespective of treatment, although eligibility criteria for the RCT were infarction of more than 2/3 of the MCA territory. However, as the individual factors promoting infarct swelling are not well understood yet, not considering these patients may possibly exclude a subgroup of patients that would have the potential to benefit from a certain treatment option in the future. The limited number of eligibility criteria are easy to implement even in small hospitals with limited staff and will most likely result in a patient mix representative of clinical practice. Conservatively treated patients are followed simultaneously and constitute the control group, which may be compared to surgically treated patients in this parallel design. Due to treatment bias it may be possible that treatment groups show significantly different baseline data making a true comparison difficult. The expected number of patients should, however, be large enough to compensate for lower event rates or differences in baseline data.

The primary outcome measure is functional outcome after 1 year determined by the mRS score and dichotomized between 0–4 versus 5–6. This endpoint was chosen to ensure comparability with the results of the pooled analysis of the RCT. Secondary outcome measures were chosen following the aforementioned open questions that could not be answered in the RCT or have not been conclusively answered in clinical studies yet. They furthermore allow subgroup analyses, e.g. elderly patients, dominant-hemispheric infarction, and timing of treatment, some of which are currently under investigation in accompanying studies [25].

The assessment of prognostic factors will lead to a better understanding of the risk of malignant infarct swelling and will aid clinical decision-making. We do not claim to assess causative associations in a cohort study. However, we expect to find predictive associations that will be of value in the immediate treatment process as well as in raising new hypotheses for subsequent treatment studies [26].

## The destiny-R investigators

### Executive committee

The *Steering Committee* is constituted of the study coordinator E. Juettler, an expereinced epidemiologist (P.U. Heuschmann), the neurosurgical principle investigators (R. Goldbrunner, C. Strauss, and P. Vajkoczy), and the neurologic principal investigators (M. Endres, W.R. Schäbitz, S. Schwab and T. Steiner).

### Assembly of investigators

Sächsisches Krankenhaus Arnsdorf (T. Back, H. Braun), Klinikum Augsburg (M. Naumann, F. Joachimski), Charité - Universitätsmedizin Berlin (E. Jüttler, H. Neugebauer, J. Witsch), Unfallkrankenhaus Berlin, Dept. of Neurology (I. Schmel, S. Kinze), Unfallkrankenhaus Berlin, Dept. of Neurosurgery (U. Meier, J. Lemke), Universitätsklinikum Carl Gustav Carus Dresden an der Technischen Universität Dresden (H. Reichmann, H. Schneider), Städtisches Krankenhaus Dresden Neustadt (J. Machetanz), Universitätsklinikum Düsseldorf (S. Jander, C. Seifert), Klinikum Duisburg (M. Scholz, E. Kigadye, S. Gillner), Klinikum Emden Hans-Susemihl-Krankenhaus (T. Büttner, M. Bauerle), HELIOS Klinikum Erfurt (A. Steinbrecher, Y. Guth), Universitätsklinikum Erlangen (S. Schwab, H. Huttner, E.M. Hauer, R. Sauer), Krankenhaus Nordwest Frankfurt (U. Meyding-Lamadé, C. Schwark), Universitätsklinik Freiburg (C. Weiller, W.D. Niesen), Krankenhaus Martha-Maria Halle-Dölau (F. Hoffmann, A. Kraft), Universitätsklinikum Halle (Saale) (S. Zierz, K. Wartenberg, K. Targan), Universitätsklinikum Hamburg-Eppendorf (C. Gerloff, G. Thomalla, C. Beck), Medizinische Hochschule Hannover (R. Dengler, K. Weißenborn, D. Milani, T. Pasedag), Universitätsklinik Heidelberg Neurologische Klinik (W. Hacke, H. Amiri), Universitätsklinikum Jena (O. Witte, A. Günther), Uniklinik Köln (GR. Fink, C. Dohmen, C. Kowoll), Universitätsklinikum Leipzig (J. Claßen, D. Schneider), Universitätsmedizin der Johannes Gutenberg-Universität Mainz (F. Zipp, B. Aral-Becher, K. Gröschel), Klinikum Meiningen (G. Heide), Johannes Wesling Klinikum Minden (P. Schellinger, H. Wuttig), St. Josef Krankenhaus Moers (HW Scharafinski, A. Hofmann), Ludwig-Maximilians-Universität München (M. Dieterich, T. Pfefferkorn), Klinikum rechts der Isar Technischen Universität München Neurokopfzentrum (B. Hemmer, H. Poppert), Universitätsklinikum Münster (B. Ringelstein, M. Ritter, R. Dziewas), Klinikum Nürnberg Süd, Dept. of Neurology (F. Erbguth, W. Dietrich), Klinikum Nürnberg Süd, Dept. of Neurosurgery (H.H. Steiner), Katholischen Kliniken Oberhausen (CW. Zimmermann, B. Fauser), Evangelisches Krankenhaus Oldenburg (T. Kretschmer, Y. Mondorf), Ernst von Bergmann Klinikum Potsdam (W. Christe, J. Schultze-Amberger), Kreiskrankenhaus Prignitz (M. Petrick), Universitätsklinikum Regensburg (U. Bogdahn, F. Schlachetzki), Klinikum Rosenheim (H. Lohner), Christian-Doppler-Klinik Salzburg, Universitätsklinikum der PMU, Dept. Of Neurology (E. Trinka, JS. Mutzenbach, G. Kalss), Klinikum Stuttgart, Dept. of Neurology (H. Bäzner, E. Schmid), Klinikum Stuttgart, Dept. of Neurosurgery (N. Hopf, C. Musahl), Eberhard Karls Universität Tübingen (A. Melms, J. Erharhaghen), Universitätsklinikum Ulm (A. Ludolph, K. Knauer), Ammerland-Klinik Westerstede (S. Kotterba, M. Teepker).

### IGNITE study group: Initiative of German NeuroIntensive Trial Engagement - Sektion Klinische Studien in der Neurointensivmedizin innerhalb der DGNI

Charité- University Medicine Berlin, Dept. of Neurology (E. Juettler, J. Witsch and M. Köhnlein); Charité- University Medicine Berlin, Dept. of Neurosurgery (S. Wolf); University of Cologne, Dept. of Neurology (C. Dohmen and C. Kowoll); University of Cologne, Dept. of Neurology (M. Reiner); University of Dresden, Dept. of Neurology (H. Schneider); University of Dresden, Dept. of Neurosurgery (T. Juratli); University of Erlangen, Dept. of Neurosurgery (D. Staykov); University of Frankfurt, Dept. of Neurology (C. Foerch); University of Giessen, Dept. of Neurology (T. Gerriets, I. Schirotzek and T. Schmelzer); University of Freiburg, Dept. of Neurology (J. Bardutzky and WD. Niesen); University of Halle, Dept. of Neurology (K. Wartenberg); University of Heidelberg, Dept. of Neurology (J. Bösel and J. Diedler); University of Heidelberg, Dept. of Neurosurgery (B. Orakcioglu); University of Jena, Dept. of Neurology (A. Günther); University of Leipzig, Dept. of Neurology (C. Hobohm); University of Munich (LMU), Dept. of Neurology (T. Pfefferkorn and M. Klein); Munich, Media consulting (N. Meckel); University of Münster, Dept. of Neurology (R. Dziewas, J. Minnerup and C. Steidl); Kantonspital St. Gall, Switzerland, Dept. of Neurosurgery (D. Engel); University of Tübingen, Dept. of Neurosurgery (M. Schuhmann and M. Skardelly); University of Würzburg, Dept of Neurosurgery (J. Eriskat and E. Kunze).

## Abbreviations

DESTINY-R: DEcompressive Surgery for the Treatment of malignant INfarction of the middle cerebral arterY - Registry; RCT: Randomized controlled trial; MCA: Middle cerebral artery; DHC: Decompressive hemicraniectomy; mRS: Modified Ranking scale.

## Competing interests

The authors declare that they have no competing interests. There is no external funding. The study is exclusively driven by internal means of the Center for Stroke Research Berlin (CSB), the Institute for Clinical Epidemiology and Biometry, University of Würzburg, and the participating centers.

## Authors' contributions

PH and EJ participated in the design of the study. PH performed the sample size calculation and will perform the statistical analysis. HN and EJ conceived the study protocol, participated in coordination of the study centers and their contributions to the protocol, and drafted the manuscript. PH, EJ, and HN read and approved the final manuscript. The DESTINY-R Study Centers and the IGNITE Study Group all gave valuable comments on design of the study protocol and the manuscript as well as the conduct of the study. All authors read and approved the final manuscript.

## Pre-publication history

The pre-publication history for this paper can be accessed here:

http://www.biomedcentral.com/1471-2377/12/115/prepub
